# Event-related potentials elicited by the Deutsch “high-low” word illusion in the patients with first-episode schizophrenia with auditory hallucinations

**DOI:** 10.1186/s12888-016-0747-3

**Published:** 2016-02-18

**Authors:** You Xu, Hao Chai, Bingren Zhang, Qianqian Gao, Hongying Fan, Leilei Zheng, Hongjing Mao, Yonghua Zhang, Wei Wang

**Affiliations:** Department of Clinical Psychology and Psychiatry, School of Public Health, Zhejiang University College of Medicine, Hangzhou, China; Department of Psychiatry, Second Affiliated Hospital, Zhejiang University College of Medicine, Hangzhou, China; Department of Clinical Psychology, Mental Health Center, Zhejiang University College of Medicine, Hangzhou, China

**Keywords:** Auditory hallucinations, Deutsch “high-low” word illusion, Event-related potentials, Paranoid schizophrenia

## Abstract

**Background:**

The exact cerebral structural and functional mechanisms under the auditory verbal hallucinations (AVHs) in schizophrenia are still unclear. The Deutsch “high-low” word illusion might trigger attentional responses mimicking those under AVHs.

**Methods:**

We therefore have invited 16 patients with first-episode, paranoid schizophrenia, and 16 age- and gender-matched healthy volunteers to undergo the “oddball” event-related potentials elicited by the illusion. The clinical characteristics of patients were measured with the positive and negative symptom scale.

**Results:**

Besides the longer reaction time to the illusion, the standard P2 latency was shortened, the N2 latency was prolonged, and both N1 and P3 amplitudes were reduced in patients. The P3 source analyses showed the activated bilateral temporal lobes, parietal lobe and cingulate cortex in both groups, left inferior temporal gyrus in controls, and left postcentral gyrus in schizophrenia. Moreover, the N1 amplitude was positively correlated with the paranoid score in patients.

**Conclusions:**

Our results were in line with previous neurophysiological and neuroimaging reports of hallucination or auditory processing in schizophrenia, and illustrated a whole process of cerebral information processing from N1 to P3, indicating this illusion had triggered a dynamic cerebral response similar to that of the AVHs had engaged.

**Electronic supplementary material:**

The online version of this article (doi:10.1186/s12888-016-0747-3) contains supplementary material, which is available to authorized users.

## Background

Schizophrenia is a severe mental disorder with persistent perceptual and cognitive impairments [1, 2], such as semantic processing deficits [3–5] and attention problems [6]. Auditory hallucinations especially auditory verbal hallucinations (AVHs) are one of the characteristic symptoms of the disorder, which might range from single words or phrases to voices giving commands, comments, insults, or encouragement [7]. Many attempts to understand the neural correlates of AVHs have been trialed in recent years. For instance, the AVHs, with more ambiguous or salient signals often accepted as real and meaningful, were demonstrated broadly involved in the processes of attention, cognition, and emotion of a patient with schizophrenia [8]. It has been shown that the semantic processing of auditory information contributed to the generation of AVHs in a general population [9], but this contribution has not proven in patients with schizophrenia [10].

On the other hand, some scholars consider the relationship between illusion and hallucination, and suggest that the hallucination is the illusion of the reality [11, 12]. Thus, the illusion-related processing might be used as a specific probe to study AVHs. Deutsch [13] reported a musical illusion, a dichotic listening phenomenon that happened when a participant received one sine-wave sequence from one ear and another simultaneously presented but phase-reversed sequence from the other ear, i.e., when one ear received the high tone, the other ear received the low tone and vice versa. In this case, although the high and the low tones were delivered to both ears, right-handed participants typically perceived a single low tone at one ear alternating with a single high tone at the other regardless of how the earphones are positioned [13, 14]. Later, Deutsch [15] used words, such as “high” and “low”, to replace the tones, and developed a Deutsch “high-low” word illusion. Listening to this pattern through stereo loudspeakers for a while, English-speaking people had reported hearing English words which were actually not presented, such as “buy loan”, “long time”, “no, no” and “boatman” [15]; while the healthy Chinese-speaking people and Chinese patients with cluster A personality disorders had reported hearing Chinese words which were related to their personality traits [16, 17].

The neuroimaging evidences have suggested that the abnormalities related to auditory hallucinations were in the auditory “what” pathway including anterior and posterior temporal cortices and in the “where” pathway including the superior temporal, inferior parietal and superior frontal cortices [7, 8]. For instance, the functional neuroimaging studies have associated AVHs with brain areas involved in speech generation, speech perception and auditory processing [18–20]. The areas activated during hallucinations were the temporal and prefrontal cortical areas [21, 22], or the inferior frontal/ insular, anterior cingulate, temporal as well as some subcortical regions [23, 24].

On the other hand, the electroencephalography (EEG) provides noninvasive measures with superior temporal resolution (in milliseconds) of brain activities, and it is more suitable to capture the rapidly occurring processes than the neuroimaging techniques such as fMRI or PET [25]. Using the event-related potentials (ERPs), many hallucinations related cognitive deficits in schizophrenia have been documented. The N4 (N400) potential, considered as a semantic priming related component, has been widely used to explore different aspects of language disturbance in schizophrenia [5], though it was not clear whether this component was a neurophysiological biomarker of the semantic processing dysfunction in schizophrenia [26, 27]. The N4 was more negative when examining the processes of context use while it was normal or reduced when examining the primary processes of initial activation within the semantic networks [5]. The component P3 (P300), considered as an attention-related cognitive potential, has also been trialed in illusion studies [28, 29]. In schizophrenia, the reduced and prolonged P3 component has been consistently observed [30–32], which was also related to the auditory hallucination and clinical symptom severities [33, 34]. More specifically, a left-lateralized reduction of P3 component which was closely related to the decrease of the normal left-dominance function of processing syllables and complex tones [35], was also related to the impairments of the temporal lobe in schizophrenia [34, 36, 37]. Therefore, P3 potential might be one of the endophenotypic markers of schizophrenia [38], and might help to disclose the mechanisms underlying AVHs.

However, due to the difficulty to employ on-going hallucinations (such as AVHs) to trigger an ERP study, there is no investigation illustrating the dynamic changes under AVHs up to date, for instance, in a time window of N1-P3 components. Moreover, since the various AVH contents [7] could hardly be used to trigger an ERP, we would like to adopt the Deutsch “high-low” word illusion as a stimulus in the present study, expecting that the word illusion triggered ERPs indicate the specific pathology of AVHs in schizophrenia. Therefore, we have hypothesized that 1) patients with schizophrenia have a reduced P3 over the scalp topography and 2) there is a prominent cerebral generator responsible for the word illusion processing.

## Methods

### Participants

Sixteen healthy participants were recruited from medical staff and the community. They were physically healthy, did not suffer from any psychiatric or neurological disorders. Sixteen patients were diagnosed as having paranoid schizophrenia with auditory hallucinations in the acute phase according to the criteria of the International Classification of Diseases-10 [39] after a semistructured clinical interview by two experienced psychiatrists (LZ & WW) separately. All patients had experienced their first acute episode with relatively short disease durations, and displayed moderate to low levels of psychopathology as accessed using the Positive and Negative Syndrome Scale (PANSS) [40] (Table 1). All participants were confirmed to have no other confounding factors including affective or schizoaffective disorder, nor prior history of head injury, alcohol or tobacco abuse, psychoactive substance abuse, central nervous system inflammation, nor neurocognitive or other disorders influencing the decisional capacity (understanding, appreciation, reasoning, and expression of choice of an action) through the semistructured clinical interview. The demographic data, medical history, and medication information were listed in Table 1. There was no age (*t* = −1.33, *p* = 0.20) or gender (*χ*^2^ = 2.032, *p* = 0.15) difference between groups. Six patients were medication free and the remaining patients had been treated with atypical antipsychotics at regular doses for no more than 2 weeks (also see Table 1). A recent CT or MRI scan was available in order to ensure that all patients were free from any organic brain lesions. There was no statistically significant difference in educational level between the two groups. All participants were extreme right-handers according to a Chinese translation of the Edinburgh Handedness Scale [41]. The study was approved by the Ethics Committee of Zhejiang University College of Medicine (No. ZGL201307-2-1). Two PhD candidates (YX & HC) were available to explain the written informed consent by presenting a Powerpoint presentation, showing a hypothetical EEG experiment onsite, and showing a signed written informed consent to the participants or their next of kin. YX and HC were also available to aid in the proper filling of the required demographic information, questionnaire and the informed consent, and to ensure corrective feedbacks. In particular, all patients were ensured to have a free expression of choice, and to fully understand the study protocol information (i.e., its atraumatic features, and its usefulness for scientific research and for disease understanding). These patients had to repeat the consent information orally to one experienced psychiatrist (from ZL & WW) and one PhD candidate (YX & HC). All adult participants gave their written informed consent to participate. For patients of 16–17 years old, we have obtained the written informed consent by their next of kin, through a surrogate consent procedure, regarding participating in our study. The study also conformed to the Helsinki Declaration concerning human rights and informed consent and followed correct procedures concerning treatment of humans in research.

**Table 1:** Demographic data in patients with schizophrenia (*n* = 16) and healthy volunteers (*n* = 16)

	Schizophrenia	Controls
Gender (f/m)	5/11	9/7
Age (in years; mean ± SD/ range)	22.4 ± 5.8/16–36	20.4 ± 1.6/19–25
Positive and negative syndrome scale (mean ± SD)		
Total	59.6 ± 13.7	–
Positive scale	14.4 ± 4.4	–
Negative scale	9.8 ± 3.7	–
General psychopathology scale	31.2 ± 9.4	–
Lack of action	4.63 ± 1.78	–
Thinking disorder	9.31 ± 2.98	–
Irritation	5.69 ± 2.12	–
Paranoid	4.81 ± 1.38	–
Depression	9.81 ± 2.81	–
Others	9.38 ± 2.50	–
Disease history (in months)	2.1 ± 0.7	–
Medication		
olanzapine	6	–
risperidone	3	–
quetiapine	1	–

### Stimuli and procedure

Participants were seated in an armchair in a quiet and dimly lit room while stimuli were delivered according to the “oddball” paradigm through headphones at an inter-stimulus interval randomized from 1500-2000 ms. The target stimulus was a 250 ms segment (a full circle) of the “high-low” word illusion [15] and the standard stimulus was a 1000 Hz tone with the same duration, both edited by Adobe Audition (Adobe Systems Incorporated). The standard stimuli were delivered 160 times (80 %) while the target stimuli were 40 times (20 %) in a randomized order. After a short duration of training, participants were asked to respond to the target stimuli, by pressing a button using the index finger of their artful hand as soon as possible, and their reaction times to the target were recorded. The reaction accuracies for the two groups were all nearly 100 % due to the simplicity of the task.

### ERP recording

The EEG recording were performed with 32 electrodes embedded in an electro-cap (Electro-Cap International, Inc.) according to the 10–20 International System and intermediate positions (Fp1, Fpz, Fp2, F3, F4, F7, F8, Fz, Fc1, Fc2, Fc5, Fc6, T7, T8, C3, C4, Cz, Cp1, Cp2, Cp5, Cp6, P3, P4, P7, P8, Pz, O1, Oz, O2, POz, M1, and M2). Recording was made with the average reference. The EEG was amplified by an ANT amplifier (Enschede, the Netherlands) and the impedance of all electrodes was kept below 10 kΩ. The EEG was continuously recorded with a sampling rate of 1024 Hz and then re-referred to the average activity of the two mastoid electrodes (M1 and M2) off-line. Trials containing electrooculogram (EOG) and other artefacts were eliminated by ASA software (ANT software, version 4.7., The Netherlands) using a principal component analysis method that models the brain signal and artefact subspaces [42]. Data were filtered with a bandpass of 0.1-30 Hz. Epochs beginning 100 ms prior to stimulus onset and continuing for 900 ms were created and segments of the record contaminated by artefacts (±70 μv) were rejected from averaging.

ERPs were analyzed in terms of peak latency and baseline-to-peak amplitude of the respective maximal deflections in the following time windows: 50–150 ms and 100–300 ms for standard N1and P2 respectively; 50–150 ms, 100–200 ms, 150–250 ms, 250–400 ms for target N1, P2, N2 and P3 respectively.

### Source analyses

The statistical parametric mapping (SPM 8, http://www.fil.ion.ucl.ac.uk/spm) toolbox for M/EEG data was used for source analyses of the N1, P2, N2 and P3 components elicited by word illusion segment (i.e., the target) in both groups. The SPM 8 provides “source reconstruction resulting in a spatial projection of sensor data into brain space and considers brain activity as comprising a very large number of dipolar sources spread over the cortical sheet based on Bayesian inversion of hierarchical Gaussian process models” [43–45]. In the present study, the SPM default template head model (normal cortical mesh sizes, i.e., 8196 vertices were used for calculating) based on the Montreal Neurological Institute (MNI) brain was used, and the multiple sparse priors algorithm [43] was applied to the time window of each component (again, 50–150 ms, 100–200 ms, 150–250 ms, 250–400 ms for target N1, P2, N2 and P3 respectively; re-referred to average reference) for the source reconstruction, as this method gives the most plausible results and has greater model evidence [46]. After the reconstruction, a source level statistical analysis based on the random field theory [44, 47] was also performed to detect any significant difference P3 sources between groups.

### Statistical analyses

The mean age and reaction time in the two groups were analyzed by the independent *t* test. The mean latency/ amplitude of standard N1, P2 and target N1, P2, N2, and P3 at nine electrode sites (F3, Fz, F4, C3, Cz, C4, P3, Pz, and P4) were analyzed using a four-way ANOVA, i.e., Group (2) X Gender (2) X Sagittal Positions (3: frontal, central, and parietal) X Lateral Positions (3: left, central, and right) with the post-hoc Bonferroni test. The mean latency/amplitude difference between two groups at each site were analyzed by the independent *t* test. The Pearson correlation was applied between latency/amplitude of target N1, P2, N2 and P3 at each site and PANSS scale scores (i.e., total score, positive scale, negative scale, general psychopathology scale, lack of action, thinking disorder, irritation, paranoid, depression, others) in schizophrenia group, while the relationship would not be recognized unless it was significantly at three adjacent sites. The alpha level of significance was set at .05. Based on the design of detecting a group difference in one ERP parameter, for example, the statistical power of our study reached .89 for raw, .95 for column, and .52 for interaction effects.

## Results

**Fig. 1:**
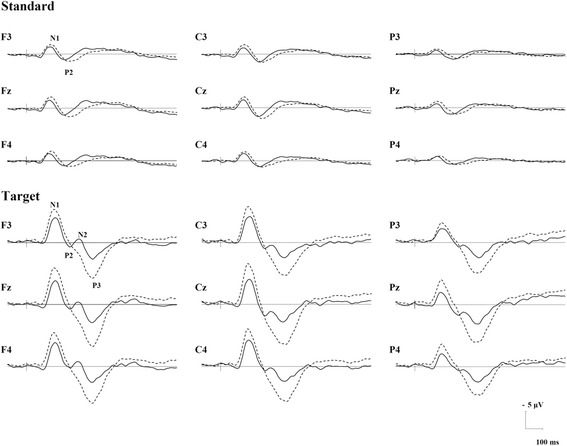
ERP grand averages in controls and schizophrenia at nine electrode sites. Controls are presented in dashed (*n* = 16), and schizophrenia in solid (*n* = 16) lines

The mean reaction time was significantly prolonged (*t* = −2.51, *p* = 0.02) in patients with schizophrenia (384.6 ms ± 90.7) compared to that in healthy participants (317.8 ± 55.3). Each participant clearly showed ERP traces to standard and target stimuli at all 32 electrodes. The grand averages at selected nine electrodes are shown in Fig. 1. Some detailed data, such as the mapping of each potential component, is omitted, for the sake of data presentation brevity.

### Morphology triggered by standard stimuli

In each participant, the N1 and P2 to the standard stimuli were clear.

#### N1 component

For standard N1 latencies, no significant main effect of either group (*F*(1, 28) = 1.01, *MSE* = 2073.67, *p* = 0.32), gender (*F*(1, 28) = 0.01, *MSE* = 17.30, *p* = 0.93), sagittal (*F*(2, 112) = 3.15, *MSE* = 1130.73, *p* = 0.08), or lateral (*F*(2, 112) = 0.23, *MSE* = 45.63, *p* = 0.75) was detected. For standard N1 amplitudes, no significant group (*F*(1, 28) = 1.60, *MSE* = 52.89, *p* = 0.22) effect was detected, but main effect of sagittal (*F*(2, 112) = 42.39, *MSE* = 122.34, *p* < 0.01), lateral (*F*(2, 112) = 6.33, *MSE* = 6.88, *p* < 0.01), and gender (*F*(1, 28) = 6.09, *MSE* = 201.08, *p* = 0.02) were all significant. The standard N1 amplitudes were more pronounced at frontal (−3.9 μV ± 0.4, *p* < 0.01, 95 % CI [1.2, 2.6]) and central (−3.6 μV ± 0.4, *p* < 0.01, 95 % CI [1.0, 2.0]) sites compared with those at parietal (−2.1 μV ± 0.3); at central (−3.3 μV ± 0.4) sites compared with those at left (−2.9 μV ± 0.3, *p* < 0.01, 95 % CI [0.1, 0.8]); and in males (−4.1 μV ± 0.5) compared with those in females (−2.3 μV ± 0.5, *p* = 0.02, 95 % CI [0.3, 3.2]).

#### P2 component

For standard P2 latencies, there was significant main effect of group (*F*(1, 28) = 9.81, *MSE* = 62121.12, *p* < 0.01), with shortened P2 latencies in schizophrenia (204.1 ms ± 7.2) compared to that in controls (234.7 ms ± 6.7, 95 % CI [10.6, 50.7]). Main effect of the gender (*F*(1, 28) = 0.54, *MSE* = 3392.27, *p* = 0.47), sagittal (*F*(2, 112) = 0.68, *MSE* = 235.13, *p* = 0.51), or lateral (*F*(2, 112) = 2.90, *MSE* = 830.89, *p* = 0.08) was not significant. For standard P2 amplitudes, no significant main effect of group (*F*(1, 28) = 0.23, *MSE* = 3.18, *p* = 0.63) or gender (*F*(1, 28) = 2.30, *MSE* = 31.63, *p* = 0.14) was detected, but of sagittal (*F*(2, 112) = 6.00, *MSE* = 10.19, *p* < 0.01) and lateral (*F*(2, 112) = 11.09, *MSE* = 9.98, *p* < 0.01) sites were all significant. The standard P2 amplitudes were more pronounced at central sites (2.5 μV ± 0.3) compared with those at frontal (1.9 μV ± 0.3, *p* < 0.01; 95 % CI [0.2, 1.1]) and parietal (2.0 μV ± 0.2, *p* = 0.03; 95 % CI [0.1, 1.0]); at central (2.5 μV ± 0.3) sites compared with those at left (2.0 μV ± 0.2, *p* < 0.01; 95 % CI [0.2, 0.8]) and right (1.8 μV ± 0.2, *p* < 0.01; 95 % CI [0.3, 1.0]).

### Morphology triggered by target stimuli

In each participant, the N1, P2, N2, P3 (but not N4) to the target stimuli were clear.

#### N1 component

For target N1 latencies, there was no significant main effect of group (*F*(1, 28) = 2.52, *MSE* = 8990.30, *p* = 0.12), sagittal (*F*(2, 112) = 2.89, *MSE* = 423.23, *p* = 0.06) or lateral (*F*(2, 112) = 0.31, *MSE* = 34.37, *p* = 0.74) either, but significant gender effect (*F*(1, 28) = 6.65, *MSE* = 23685.54, *p* = 0.02) was found, with prolonged N1 latencies in females (152.3 ms ± 5.6; 95 % CI [3.9, 34.0]) compared with those in males (133.4 ms ± 4.8). For target N1 amplitudes, significant main effects of group (*F*(1, 28) = 6.81, *MSE* = 627.10, *p* = 0.01) and of sagittal sites (*F*(2, 112) = 40.88, *MSE* = 612.90, *p* < 0.01), but not of gender (*F*(1, 28) = 0.20, *MSE* = 18.29, *p* = 0.66) or of lateral sites (*F*(2, 112) = 3.02, *MSE* = 32.24, *p* = 0.08) were detected. The target N1 amplitudes were more pronounced at frontal (−11.1 μV ± 0.8, *p* < 0.01; 95 % CI [2.7, 6.0]) and central (−11.5 μV ± 0.8, *p* < 0.01; 95 % CI [3.3, 6.3]) sites compared with those at parietal (−6.8 μV ± 0.5), and was decreased in schizophrenia (−8.2 μV ± 0.9) compared with those in controls (−11.3 μV ± 0.8; 95 % CI [0.7, 5.5]) (Table 2).

**Table 2:** Latencies and amplitudes of ERP components to target stimuli in controls and schizophrenia

Site	N1			
	Latency		Amplitude	
	Controls	Schizophrenia	Controls	Schizophrenia
F3	139.45 ± 21.76	141.46 ± 14.05	−13.48 ± 7.99	−9.18 ± 3.42
Fz	140.91 ± 21.14	147.02 ± 27.97	−12.81 ± 5.02	−8.63 ± 3.89*
F4	140.24 ± 19.10	143.72 ± 20.69	−11.50 ± 4.09	−8.55 ± 4.62
C3	139.14 ± 21.19	146.22 ± 26.66	−12.60 ± 4.41	−9.96 ± 3.38
Cz	137.74 ± 17.17	144.21 ± 27.19	−14.00 ± 6.06	−9.52 ± 3.60*
C4	138.35 ± 16.93	144.27 ± 29.15	−12.18 ± 4.60	−9.18 ± 3.57*
P3	132.19 ± 17.84	140.55 ± 30.68	−7.27 ± 2.79	−5.74 ± 2.58
Pz	133.53 ± 18.37	141.77 ± 35.92	−9.04 ± 3.45	−5.34 ± 2.65**
P4	136.40 ± 19.74	139.88 ± 27.93	−7.42 ± 2.55	−4.93 ± 2.74*
	P2			
	Latency		Amplitude	
	Controls	Schizophrenia	Controls	Schizophrenia
F3	220.48 ± 27.61	225.81 ± 13.90	0.29 ± 4.57	1.84 ± 4.76
Fz	218.95 ± 27.64	225.57 ± 21.76	0.56 ± 6.98	2.15 ± 5.40
F4	219.38 ± 28.54	225.45 ± 21.93	0.48 ± 5.78	0.83 ± 4.49
C3	223.71 ± 28.28	229.54 ± 16.73	1.51 ± 6.63	2.85 ± 5.90
Cz	223.35 ± 33.24	229.05 ± 21.91	3.72 ± 10.13	4.75 ± 7.85
C4	226.28 ± 33.57	229.60 ± 21.26	1.69 ± 6.86	1.97 ± 5.33
P3	218.77 ± 29.20	230.63 ± 16.16	3.06 ± 4.93	2.71 ± 4.88
Pz	218.77 ± 28.97	223.92 ± 21.44	4.84 ± 6.91	5.33 ± 4.61
P4	222.98 ± 31.98	225.38 ± 22.96	2.50 ± 5.00	2.59 ± 5.19
	N2			
	Latency		Amplitude	
	Controls	Schizophrenia	Controls	Schizophrenia
F3	243.83 ± 38.62	269.53 ± 22.37*	−0.88 ± 4.58	−2.39 ± 5.65
Fz	243.40 ± 37.07	270.57 ± 20.78*	−1.23 ± 7.39	−2.01 ± 6.81
F4	245.66 ± 35.07	267.82 ± 25.89	−0.64 ± 6.02	−2.90 ± 6.69
C3	242.79 ± 38.83	268.98 ± 23.84*	0.81 ± 6.42	−0.08 ± 4.73
Cz	243.40 ± 38.78	272.89 ± 31.77*	2.67 ± 9.83	1.09 ± 7.51
C4	241.88 ± 35.60	265.32 ± 23.57*	1.32 ± 6.68	−0.62 ± 5.65
P3	242.97 ± 39.60	266.05 ± 22.88	2.58 ± 5.54	0.12 ± 4.59
Pz	246.45 ± 35.50	269.96 ± 20.53	3.97 ± 7.69	2.08 ± 4.10
P4	244.19 ± 39.32	264.35 ± 25.74	2.01 ± 5.50	0.25 ± 5.05
	P3			
	Latency		Amplitude	
	Controls	Schizophrenia	Controls	Schizophrenia
F3	340.39 ± 19.76	333.29 ± 24.25	10.71 ± 9.06	4.85 ± 5.30*
Fz	334.29 ± 21.32	335.00 ± 24.84	14.19 ± 6.63	6.10 ± 6.37**
F4	336.79 ± 20.98	335.42 ± 29.35	12.84 ± 5.12	5.50 ± 6.12**
C3	330.57 ± 28.50	324.07 ± 26.16	11.88 ± 5.81	4.18 ± 4.05**
Cz	323.36 ± 36.69	320.84 ± 27.69	14.54 ± 7.98	5.61 ± 4.60**
C4	328.80 ± 31.31	329.26 ± 32.19	12.88 ± 5.58	4.96 ± 4.43**
P3	325.26 ± 29.55	328.16 ± 23.97	11.07 ± 4.13	4.70 ± 4.84**
Pz	322.14 ± 31.82	328.47 ± 30.16	13.67 ± 5.46	6.16 ± 4.00**
P4	330.08 ± 27.20	330.24 ± 34.17	10.81 ± 4.49	5.18 ± 3.67**

*p < .05, **p < .01 vs controls; sample sizes of controls and schizophrenia were both 16. Latencies (in ms) and amplitudes (in μV) were presented as mean ± *SD*

#### P2 component

For target P2 latencies, no significant effect of group (*F*(1, 28) = 1.21, *MSE* = 5772.50, *p* = 0.28), gender (*F*(1, 28) = 4.00, *MSE* = 19091.32, *p* = 0.06), sagittal (*F*(2, 112) = 3.16, *MSE* = 698.30, *p* = 0.06) or lateral (*F*(2, 112) = 0.48, *MSE* = 46.24, *p* = 0.55) was detected (Table 2). For target P2 amplitudes, main effect of group (*F*(1, 28) =0.24, *MSE* = 67.55, *p* = 0.63) or gender (*F*(1, 28) = 0.64, *MSE* = 176.07, *p* = 0.43) was not significant, while main effects of lateral (*F*(2, 112) = 7.02, *MSE* = 152.49, *p* < 0.01), and of sagittal (*F*(2, 112) = 14.50, *MSE* = 107.78, *p* < 0.01) were detected. The target P2 amplitudes were more pronounced at central sites (3.7 μV ± 1.3) compared to those at left (1.9 μV ± 0.9, *p* < 0.01; 95 % CI [0.7, 3.0]) and at right (8.9 μV ± 0.8, *p* < 0.01; 95 % CI [0.6, 2.4]), and were decreased at frontal sites (1.0 μV ± 1.0) compared to those at parietal (3.6 μV ± 1.0; 95 % CI [0.7, 4.5]) (Table 2).

#### N2 component

For N2 latencies, significant group (*F*(1, 28) = 7.59, *MSE* = 50393.81, *p* = 0.01) and gender effects (*F*(1, 28) = 4.75, *MSE* = 31539.64, *p* = 0.04) were found, with prolonged N2 latencies in females (266.0 ms ± 7.6; 95 % CI [1.3, 42.4]) compared with those in males (244.2 ms ± 6.6), and in schizophrenia (268.9 ms ± 7.3; 95 % CI [7.0, 48.2]) compared with that in controls (241.3 ms ± 6.8). There was no significant main effect of sagittal (*F*(2, 112) = 0.003, *MSE* = 0.877, *p* = 1.00), or lateral (*F*(2, 112) = 2.97, *MSE* = 249.50, *p* = 0.06) sites (Table 2). For N2 amplitudes, no group (*F*(1, 28) = 0.47, *MSE* = 146.09, *p* = 0.50) or gender (*F*(1, 28) = 0.06, *MSE* = 17.16, *p* = 0.82) effect was detected, but main effects of sagittal (*F*(2, 112) = 11.72, *MSE* = 267.57, *p* < 0.01), and of lateral (*F*(2, 112) = 8.15, *MSE* = 58.22, *p* < 0.01) sites were significant. The N2 amplitudes were more pronounced at frontal (−1.5 μV ± 1.2) compared with those at central (1.0 μV ± 1.3, *p* < 0.01; 95 % CI [0.6, 4.3]) and parietal (1.8 μV ± 1.0, *p* < 0.01; 95 % CI [1.2, 5.5]) sites, were decreased at central (1.4 μV ± 1.3, *p* < 0.05) compared with those at left (0.0 μV ± 0.9, *p* = 0.01; 95 % CI [0.2, 2.6]) and right (0.0 μV ± 1.0, *p* < 0.01; 95 % CI [0.3, 2.4]) sites (Table 2).

#### P3 component

For P3 latencies, no significant effect of group (*F*(1, 28) = 0.01, *MSE* = 62.79, *p* = 0.92), gender (*F*(1, 28) < 0.01, *MSE* = 0.06, *p* = 1.00) or lateral (*F*(2, 112) = 2.87, *MSE* = 581.59, *p* = 0.07) was detected, but significant main effect of sagittal (*F*(2, 112) = 7.58, *MSE* = 3357.89, *p* < 0.01) was detected, with prolonged latencies at front sites (335.9 ms ± 4.2) compared with those at central (326.0 ms ± 5.5, *p* = 0.01; 95 % CI [2.3, 17.7]) and parietal (326.9 ms ± 5.3, *p* = 0.03; 95 % CI [0.7, 17.4]) sites (Table 2).

For P3 amplitudes, main effects of group (*F*(1, 28) = 20.29, *MSE* = 3638.96, *p* < 0.01) and lateral sites (*F*(2, 112) = 10.98, *MSE* = 158.89, *p* < 0.01) were detected, while the main effects of gender (*F*(1, 28) = 1.54, *MSE* = 276.17, *p* = 0.23) and of sagittal sites (*F*(2, 112) = 0.13, *MSE* = 4.21, *p* = 0.80) were not significant. The P3 amplitudes were more pronounced at central sites (10.4 μV ± 1.0) compared to those at left (8.0 μV ± 0.9, *p* < 0.01; 95 % CI [0.9, 3.8]) and at right (8.9 μV ± 0.8, *p* < 0.01; 95 % CI [0.6, 2.4]), and were also decreased in schizophrenia (5.3 μV ± 1.2) compared to those in controls (12.8 μV ± 1.1; 95 % CI [4.0, 10.8]) (Table 2).

### Source analyses

The source reconstruction of N1, P2 and N2 elicited by word illusion segments showed maximal activated areas at right parahippocampal gyrus (MNI coordinates: x = 29, y = −10, z = −14; Brodmann area 28), right fusiform gyrus (x = 46, y = −30, z = −21; Brodmann area 20) and right insula (x = 43, y = 9, z = 7; Brodmann area 13) respectively in controls, while at right fusiform gyrus (x = 46, y = −30, z = −21; Brodmann area 20), right parahippocampal gyrus (x = 29, y = −10, z = −14; Brodmann area 28) and left fusiform gyrus (x = −44, y = −32, z = −21; Brodmann area 20) respectively in schizophrenia.

**Fig. 2:**
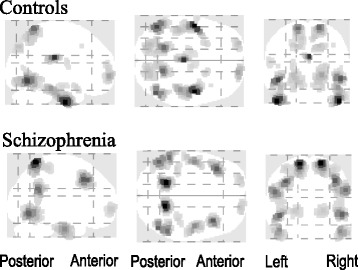
P3 source reconstruction (maximum intensity projections) in controls and schizophrenia

**Fig. 3:**
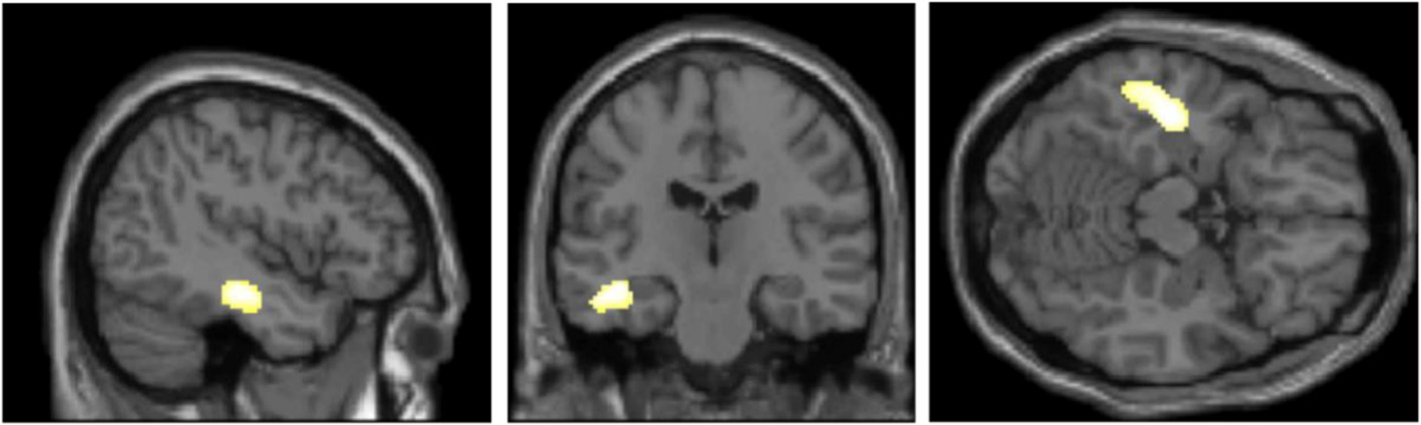
Source level statistical parametric maps displaying decreased P3 activations in schizophrenia vs controls. Significant P3 source reductions were at left inferior temporal cortex [MNI coordinates: x = −44, y = −2, z = −34, *t* = 3.14, *p* = 0.002]

The source reconstruction of P3 elicited by word illusion segments showed activated areas included bilateral temporal lobes, parietal lobe and cingulate cortex in both groups (Fig. 2). Specifically, the P3 source having maximal activity was at left inferior temporal gyrus (MNI coordinates: x = −50, y = −7, z = −41; Brodmann Area 20) in controls and at left postcentral gyrus (x = −17, y = −56, z = 67; Brodmann area 7) in schizophrenia. Moreover, the source level statistical analyses showed a significant reduction of activity (p < 0.01) in left inferior temporal cortex in schizophrenia compared with that in controls (Fig. 3).

### Relationship between ERP and clinical data

The correlation test showed some associations between N1 amplitude and the PANSS scales (for detailed data, please see Additional file 1), but only paranoid scale score was significantly correlated with N1 amplitude at parietal area (P3, *r* = .66, *p* = 0.005; Pz, *r* = .77, *p* = 0.001; P4, *r* = .618, *p* = 0.011) according to our ad hoc criteria. The scores of positive scale and thinking disorder, which contain the items of hallucinatory behavior, were not related to any ERP components (including those to the standard stimuli).

## Discussion

As hypothesized, we have found that the P3 elicited by the Deutsch “high-low” word illusion was significantly reduced and its maximal source located at a different brain area in patients with schizophrenia than those in the healthy volunteers in the present study. In combination with the decreased N1 and prolonged N2 components in schizophrenia, the findings suggested an impaired processing of the stimulus within a very short time window which might be linked with the pathology in AVHs. This is the first study to illustrate the dynamic changes to an illusion, in a time window of N1-P3, in schizophrenia.

The reaction times to the target stimuli were prolonged in schizophrenia compared with those in controls, which was consistent with previous studies [33] on the one hand. Together with reduced P3, the prolongation indicated a breakdown in the preparatory brain state which was critical for stimulus processing and later motor execution [48] in schizophrenia on the other hand.

A frontal-central scalp distribution of the standard/target N1, a more central standard/target P2, and a frontal-bilateral distributed N2 were also consistent with the previous documentation [49–52]. The reduced auditory target N1 has been reported in schizophrenia previously [49, 50]. The lowered N1 was thought to reflect the impaired ability to filter out irrelevant information in patients with schizophrenia [53, 54], as well as the lowered competition ability between auditory probes and hallucinations for auditory resources in psychiatric patients [25]. Moreover, as an exogenous component representing the early auditory processing, the N1 reduction also reflects the abnormalities in frontal-temporal lobe [55]. Similarly, the shortened standard-P2 latencies in patients replicated previous findings [52, 56, 57], indicating a faster processing speed for non-targets [56], which might reflect the impaired attention shifting to task-irrelevant stimuli. The prolonged N2 latencies in patients also replicated the results in the first-episode schizophrenia [58], which indicated a delayed stimulus-classification time in this pathology.

Similar to the scalp-distribution of the classical P3 [59], the P3 in our study was midline-distributed. The P3 reduction in our schizophrenia group was also consistent with previous researches [30–32], indicating a high-level, attention-dependent cognitive deficit when discriminating stimuli in the disorder [60]. This impairment of attentional allocation was associated with left temporoparietal cortices, which was involved in auditory-verbal imagery monitoring as demonstrated in schizophrenia [61]. Ford et al. [25] also interpreted the diminished classical P3 in patients with schizophrenia with auditory hallucinations as they preferentially attended to voices through the internal auditory channels, resulting in insufficient cortical resources to process an external stimulus.

When processing the Deutsch “high-low” word illusion, both our groups displayed activated areas of the bilateral temporal lobe, parietal lobe and cingulate cortex. Although the activation in schizophrenia was not specific, previous results have demonstrated that patients with schizophrenia with persistent hallucinations including AVHs exhibited grey matter volume decrements in the left or bilateral inferior temporal gyrus [62, 63], and their dysfunctions in speech or AVHs generation [18]. Moreover, an increased activity in the same area was found in schizophrenia patients prone to auditory hallucinations [64]. These investigations suggested that the impairment might be due to the endogenous cortical activity which impeded the processing of external stimulus.

In healthy participants, the left inferior temporal gyrus is involved in the language and semantic memory processing [65], such as the mental imagery tasks using linguistic cues [66], the mental recall of words [67], and the word generation test [68, 69]. Our finding that the left inferior temporal gyrus was activated in the healthy volunteers might be explained by these studies. In addition, it might disclose some mechanisms behind the illusion-triggered word-generating processes in normal people [17].

In schizophrenia, the activity at postcentral gyrus was reported in patients performing auditory oddball task [70], and the activity in similar brain area was documented in patients involved in the auditory verbal imagery test [71]. These activiations were associated with the somatic [72] and auditory [73] hallucination processing, and even consistently with the processing of AVHs in schizophrenia [20, 74, 75]. Another recent study on the impaired attentional modulation in the first-episode psychosis, where patients misidentified their own speech as of others, also has shown an activation of the left postcentral gyrus when patients were judging the speech to be self-generated or not [76]. Falkenberg et al. [77] suggested that the activation of the postcentral gyrus implied a higher auditory processing and influenced by the attentional mechanisms. Considering the documentation, the putative P3 source at postcentral gyrus in the present study might reflect a compensatory cerebral functioning to discriminate external from internal stimulus-sources in schizophrenia. In addition, with joint independent component analysis applied in an auditory oddball paradigm integrating ERP and fMRI data, Mangalathu-Arumana et al. [78] found that the activity of the left postcentral gyrus was consistent with the relationship between mean motor response time and the P3 amplitude. In this case, the delayed reaction time to the Deutsch “high-low” word illusion in our schizophrenia group fitted nicely with the left postcentral gyrus activation for the P3 source.

The N1 amplitude at parietal area was found correlated with the paranoid scale in patients with schizophrenia, which was consistent with the finding that N1 amplitude was reduced (more positive proneness) in these patients [79], and in people with more delusion-like ideations [80]. As N1 reflected auditory detection and discrimination [81], the reduced N1 suggested that people with paranoia might not pay attention to the target stimuli. Instead, their attention was redirected toward the surrounding environment to look for the false “hidden goal” [80]. The altered attention to the Deutsch “high-low” word illusion was also in line with a behavioral finding that the paranoid personality disorder patients reported more meaningful Chinese words when listening to the illusion [17].

However, one has to bear in mind the major design flaws of our study. Firstly, we recruited 16 healthy volunteers from medical staff and community, who might not represent a general population. Our patients were with auditory hallucination experience but without onsite hallucination-attack during the test. It remains unknown how the ERPs would be in patients with schizophrenia of either hallucination-free or under hallucination-attack. Therefore, our current results cannot be generalized to other kinds of schizophrenia or mental disorders. Secondly, we did not measure personality disorder functioning styles in patients, nor did we correlate their styles with the reported meaningful Chinese words immediately after ERP tests, due to the small sample size and the small number of meaningful words reported. Thirdly, we did not use a hallucination as a target stimulus, but simply used an illusion to trigger ERPs, and we omitted to study the frequencies and contents of AVHs in our patients. Certainly, such ideas merit further investigation. Nevertheless, we found deformed ERPs and different cerebral sources to the Deutsch “high-low” word illusion in patients.

## Conclusions

Our results addressed the cognitive problems in schizophrenia relating to the illusion, thus deepened our knowledge of hallucination processing in schizophrenia. Moreover, our study had illustrated a whole process of cerebral information processing from N1 to P3, indicating this illusion had triggered a dynamic cerebral response which might be similar to that of the AVHs had engaged.

## Additional files

Additional file 1: Table S1. Pearson correlations between ERP latencies and amplitudes and PANSS scores in schizophrenia (*n* = 16). (DOCX 26 kb)

## Abbreviations

AVHs: auditory verbal hallucinations
CT: computed tomography
ERPs: event-related potentials
fMRI: functional magnetic resonance imaging
MRI: magnetic resonance imaging
ms: millisecond
PANSS: positive and negative syndrome scale
μV: microvolt
