# Supplementary material for: Event-related potentials elicited by the Deutsch “high-low” word illusion in the patients with first-episode schizophrenia with auditory hallucinations
Source: BMC Psychiatry. 2016 Feb 18;16:33. doi: 10.1186/s12888-016-0747-3 (PMC4758162; doi:10.1186/s12888-016-0747-3)
Supplement: Additional file 1: Table S1. — Pearson correlations between ERP latencies and amplitudes and PANSS scores in schizophrenia (n = 16). (DOCX 26 kb) [file 12888_2016_747_MOESM1_ESM.docx]

**Additional file 1: Table S1.** Pearson correlations between ERP latencies and amplitudes and PANSS scores in schizophrenia (*n* = 16).

| ERP component | Index | Sites | Total score | Positive scale | Negative scale | General psychopathology scale | Lack of action | Thinking disorder | irritation | paranoid | depression | others |
| --- | --- | --- | --- | --- | --- | --- | --- | --- | --- | --- | --- | --- |
| N1 | Latency | F3 | -.08 | .00 | -.29 | -.04 | -.37 | .09 | -.37 | .37 | .13 | .27 |
|  |  | Fz | .07 | .04 | -.19 | .09 | -.21 | .10 | -.23 | .40 | .30 | .31 |
|  |  | F4 | .03 | .05 | -.25 | .06 | -.28 | .12 | -.27 | .42 | .27 | .28 |
|  |  | C3 | -.05 | -.01 | -.24 | -.01 | -.26 | .05 | -.29 | .38 | .26 | .21 |
|  |  | Cz | .01 | .02 | -.26 | .06 | -.22 | .07 | -.22 | .34 | .28 | .19 |
|  |  | C4 | .04 | .08 | -.24 | .07 | -.19 | .13 | -.18 | .40 | .25 | .15 |
|  |  | P3 | .01 | .09 | -.27 | .04 | -.21 | .13 | -.17 | .40 | .28 | .25 |
|  |  | Pz | -.01 | -.03 | -.22 | .05 | -.17 | .04 | -.19 | .34 | .28 | .15 |
|  |  | P4 | -.16 | -.07 | -.20 | -.14 | -.24 | -.05 | -.38 | .15 | .18 | .03 |
|  | Amplitude | F3 | .35 | -.04 | .12 | .45 | .23 | .17 | .34 | .43 | .53* | .29 |
|  |  | Fz | .25 | -.05 | .12 | .34 | .19 | .11 | .27 | .42 | .50* | .13 |
|  |  | F4 | .36 | .04 | .22 | .41 | .31 | .17 | .36 | .45 | .49 | .08 |
|  |  | C3 | .24 | .07 | .10 | .28 | .18 | .20 | .18 | .36 | .32 | .11 |
|  |  | Cz | .31 | .01 | .23 | .35 | .23 | .16 | .31 | .40 | .39 | .26 |
|  |  | C4 | .35 | .06 | .19 | .40 | .21 | .21 | .36 | .47 | .46 | .26 |
|  |  | P3 | .49 | .39 | -.04 | .53* | .09 | .54* | .41 | .66** | .43 | .19 |
|  |  | Pz | .48 | .38 | -.08 | .52* | -.01 | .56* | .33 | .77** | .51* | .20 |
|  |  | P4 | .39 | .24 | -.11 | .44 | -.04 | .42 | .51* | .62* | .42 | .23 |
| P2 | Latency | F3 | .13 | .33 | -.09 | .08 | -.04 | .33 | .15 | .42 | -.11 | .11 |
|  |  | Fz | -.02 | .16 | -.15 | -.03 | -.10 | .14 | .14 | .20 | -.21 | .14 |
|  |  | F4 | .19 | .27 | .02 | .16 | .09 | .24 | .34 | .31 | -.01 | .11 |
|  |  | C3 | .12 | .27 | -.13 | .09 | -.07 | .26 | .22 | .39 | -.06 | .19 |
|  |  | Cz | .03 | .17 | -.10 | .01 | -.03 | .11 | .21 | .15 | -.18 | .06 |
|  |  | C4 | .04 | .20 | -.04 | -.00 | .04 | .14 | .18 | .11 | -.20 | -.07 |
|  |  | P3 | .04 | .35 | -.21 | .01 | -.06 | .31 | .36 | .16 | -.27 | -.04 |
|  |  | Pz | .25 | .45 | -.07 | .19 | .08 | .39 | .39 | .23 | -.03 | .07 |
|  |  | P4 | .13 | .38 | -.02 | .04 | .11 | .26 | .27 | .11 | -.26 | -.06 |
|  | Amplitude | F3 | .15 | -.02 | .35 | .11 | .35 | -.02 | .42 | -.10 | -.04 | .07 |
|  |  | Fz | -.06 | -.21 | .33 | -.09 | .22 | -.21 | .16 | -.12 | -.07 | .01 |
|  |  | F4 | .14 | -.07 | .29 | .13 | .23 | -.02 | .29 | .05 | .12 | .06 |
|  |  | C3 | .14 | -.01 | .41 | .07 | .34 | -.06 | .36 | -.10 | -.06 | .07 |
|  |  | Cz | .05 | -.01 | .30 | -.01 | .21 | -.05 | .29 | -.06 | -.11 | .00 |
|  |  | C4 | .11 | .05 | .23 | .06 | .14 | .04 | .35 | .00 | -.04 | .06 |
|  |  | P3 | .08 | .04 | .20 | .05 | .22 | -.00 | .41 | .10 | -.03 | -.02 |
|  |  | Pz | .05 | .03 | .22 | -.01 | .12 | -.01 | .23 | .16 | .02 | .07 |
|  |  | P4 | .19 | .15 | .22 | .14 | .16 | .12 | .40 | .22 | .06 | .14 |
| N2 | Latency | F3 | .33 | .28 | -.06 | .32 | .11 | .39 | .35 | .32 | .02 | .41 |
|  |  | Fz | .31 | .21 | -.05 | .32 | .11 | .34 | .33 | .33 | .06 | .46 |
|  |  | F4 | .22 | .23 | -.07 | .22 | .07 | .32 | .32 | .21 | -.10 | .26 |
|  |  | C3 | .35 | .31 | -.07 | .34 | .11 | .43 | .40 | .41 | .09 | .42 |
|  |  | Cz | .10 | .08 | .02 | .07 | .05 | .11 | .10 | .06 | -.09 | .29 |
|  |  | C4 | .33 | .42 | .01 | .25 | .14 | .41 | .31 | .37 | -.02 | .16 |
|  |  | P3 | .16 | .14 | .14 | .12 | .19 | .08 | .41 | .01 | -.09 | .27 |
|  |  | Pz | .33 | .45 | -.05 | .26 | .08 | .44 | .30 | .40 | .09 | .18 |
|  |  | P4 | .34 | .46 | .10 | .24 | .17 | .42 | .36 | .31 | -.05 | .12 |
|  | Amplitude | F3 | .04 | -.03 | .17 | .05 | .14 | .01 | .15 | .16 | .06 | -.20 |
|  |  | Fz | -.17 | -.15 | .08 | -.15 | -.01 | -.14 | -.00 | .06 | -.04 | -.19 |
|  |  | F4 | -.01 | -.08 | .04 | .03 | .01 | -.01 | .07 | .20 | .13 | -.11 |
|  |  | C3 | .12 | -.03 | .33 | .08 | .23 | -.03 | .27 | .06 | .00 | -.06 |
|  |  | Cz | -.02 | -.02 | .16 | -.04 | .06 | -.03 | .20 | .03 | -.07 | -.12 |
|  |  | C4 | .06 | .00 | .11 | .06 | .03 | .04 | .27 | .09 | .02 | -.02 |
|  |  | P3 | .16 | .15 | .11 | .16 | .17 | .16 | .43 | .26 | -.01 | -.12 |
|  |  | Pz | .18 | .08 | .23 | .15 | .19 | .10 | .38 | .28 | .11 | .02 |
|  |  | P4 | .17 | .11 | .14 | .16 | .14 | .12 | .46 | .23 | .07 | .11 |
| P3 | Latency | F3 | .18 | .03 | .00 | .19 | .05 | .18 | .07 | .39 | .17 | .33 |
|  |  | Fz | .07 | .18 | -.09 | .02 | -.01 | .23 | -.13 | .16 | -.10 | .13 |
|  |  | F4 | .06 | -.07 | .05 | .06 | .01 | .02 | -.06 | .16 | .09 | .25 |
|  |  | C3 | .13 | -.13 | .06 | .17 | .12 | .00 | .04 | .21 | .24 | .33 |
|  |  | Cz | .24 | -.09 | .00 | .31 | .13 | .08 | .21 | .29 | .30 | .45 |
|  |  | C4 | .10 | -.22 | .01 | .17 | .08 | -.09 | .08 | .19 | .12 | .37 |
|  |  | P3 | -.16 | -.27 | -.02 | -.12 | .01 | -.20 | -.05 | -.06 | -.10 | .13 |
|  |  | Pz | .01 | -.18 | -.04 | .06 | .04 | -.08 | .01 | .11 | .00 | .24 |
|  |  | P4 | .21 | -.04 | -.04 | .27 | .07 | .08 | .15 | .25 | .20 | .33 |
|  | Amplitude | F3 | .09 | .07 | -.14 | .11 | -.21 | .11 | .11 | .17 | .14 | -.09 |
|  |  | Fz | .14 | .05 | -.08 | .18 | -.12 | .11 | .28 | .19 | .21 | -.03 |
|  |  | F4 | .16 | .05 | -.06 | .21 | -.09 | .11 | .32 | .17 | .20 | -.02 |
|  |  | C3 | .02 | -.02 | -.20 | .08 | -.25 | .05 | .07 | .18 | .17 | -.13 |
|  |  | Cz | -.14 | -.10 | -.19 | -.07 | -.27 | -.05 | .05 | .15 | .12 | -.18 |
|  |  | C4 | .03 | .03 | -.17 | .08 | -.21 | .07 | .33 | .12 | .09 | .03 |
|  |  | P3 | .04 | .06 | -.22 | .11 | -.21 | .11 | .18 | .31 | .17 | -.17 |
|  |  | Pz | -.07 | .06 | -.24 | -.02 | -.26 | .08 | .22 | .26 | .06 | -.22 |
|  |  | P4 | -.03 | .15 | -.22 | -.01 | -.21 | .10 | .41 | .16 | .01 | -.07 |

Note: * *p* < .05, ** *p* < .01; PANSS, the positive and Negative Syndrome Scale.
